# Partnering for Change: collaborating to transform occupational therapy services that support inclusive education

**DOI:** 10.3389/fpubh.2023.1275920

**Published:** 2023-09-25

**Authors:** Wenonah Campbell, Cheryl Missiuna, Leah Dix, Sandra Sahagian Whalen

**Affiliations:** ^1^School of Rehabilitation Science, McMaster University, Hamilton, ON, Canada; ^2^CanChild Centre for Childhood Disability Research, McMaster University, Hamilton, ON, Canada

**Keywords:** inclusion, Partnering for Change, tiered services, school-based service models, occupational therapy, education

## Abstract

The United Nations champions inclusive education as a moral obligation, requiring equitable learning environments that meet all individuals’ diverse learning needs and abilities, including children and youth. Yet the practice of inclusive education is variable and implementation challenges persist. A participatory action research framework was used to develop a solution, Partnering for Change (P4C), which is a tiered service delivery model that bridges health and education by re-envisioning occupational therapy services and transforming the role of the occupational therapist from a service provider for individual children to a collaborative partner supporting the whole school community. This perspective article will describe the P4C model and its evolution, and will outline how it has been implemented in Canadian and international contexts to facilitate children’s inclusion and participation in educational settings.

## 1. Introduction

Inclusive education is a human right first endorsed in the 1989 United Nations Convention on the Rights of the Child (1). The Salamanca Statement and Framework for Action on Special Needs Education, adopted by 92 countries, reflected international acceptance of inclusive education as a right for all children (2). Inclusive education means that children with diverse abilities and circumstances receive high-quality education in general education classrooms in their neighborhood schools (3). The goals of education for all children should be participation, a sense of belonging, affirming social relationships, and positive developmental and learning outcomes (3–6). Research shows that children with disabilities who attend inclusive schools and who participate in general education classrooms do better physically, emotionally, socially, and academically than children who are in congregated settings (i.e., special education classrooms or segregated schools) (4, 7–9). In addition to the societal argument for inclusion, recent studies also show that children without disabilities have better outcomes (10). It seems clear to us that the provision of inclusive education in schools is of paramount importance for the 20% or more children who have challenges participating in daily school routines, activities, and accessing the curriculum due to a disability or impairment that impacts their neurodevelopmental, learning, or social–emotional function, and that it is also beneficial for all children (7, 11).

Yet despite inclusive education being an international goal, the adoption of fully inclusive educational practice has not been achieved (12). For example, although the Canadian government endorsed the United Nations Convention on the Rights of Persons with Disabilities (13), children with disabilities continue to face barriers to accessing educational services (14). Similar discrepancies between government policy and implementation of inclusive school cultures can be found across the United States, the United Kingdom, and other countries, with most falling short of expectations (10, 14–17).

Our multidisciplinary research team from *CanChild*, a center for childhood disability research at McMaster University in Canada, recognized that if inclusive education is the goal, a fundamental paradigm shift was needed in how rehabilitation services in schools were conceptualized (18, 19). Building from our belief about the importance of supporting all children, we rationalized that occupational therapists needed to be accountable for the participation and inclusion of all children, to connect their roles and contributions to the educational context, and to recalibrate from the traditional focus on remediation of individual children to an equity-focused, needs-based approach to service delivery that was aligned with the aims of inclusive education (18, 20). In Canada, occupational therapists working in schools have typically focused on individual children with an identified disability, conducting assessments, writing reports, and providing suggestions using a consultation model: but does this approach make any difference? Are their suggestions timely, relevant to children’s needs or the curriculum, and able to be implemented as part of teachers’ classroom routines? Our research team was aware that occupational therapists and teachers were grappling with such issues, each wondering how they could better meet the needs of children but unaware of the others’ perspectives, expertise, and skills. Our team asked – what if occupational therapists could deliver a service in which they collaborated with teachers and supported each other in complementary and synergistic ways? What would this service look like and what could this mean for inclusive education?

In 2008, our team utilized a participatory action approach and invited rehabilitation service providers, teachers, administrators, families, and representatives from the health and education sectors in Ontario, Canada to discuss long waitlists for school-based occupational therapy, uncertain outcomes, inequitable access to service for children with varied needs, and lack of progress with inclusive education (19). The participants agreed that it was time to work collaboratively to address these issues; the outcome was a model called *Partnering for Change*. Partnering for Change was more than a description of the inter-sectoral participants who were partnering together to create necessary change; it became the name of the occupational therapy service (18, 19). Interested readers can learn about our approach in depth by reading our publication describing this process (19).

## 2. What is Partnering for Change (P4C)?

**Figure 1:**
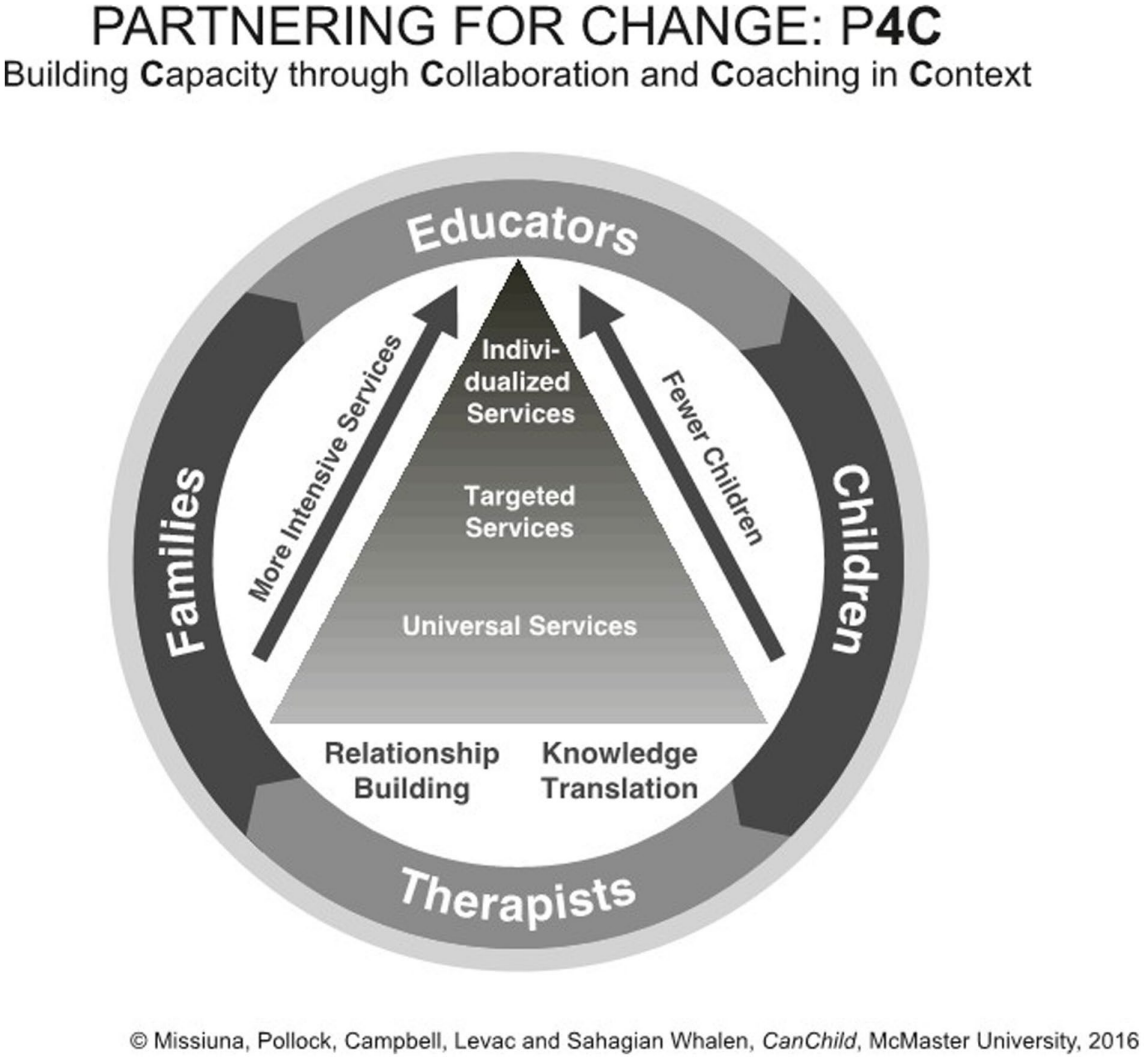
A visual depiction of the P4C Model.

Shortened to P4C, the principles of this school-based occupational therapy service involve *Partnering to Build Capacity through Collaboration and Coaching in Context*. The conceptual model developed by our team is shown in Figure 1. Italics are used in the text to further highlight the key concepts within the model, consistent with the figure. P4C is a needs-based service delivery model that emphasizes *partnerships* among occupational therapists, teachers, families, and children. Teachers are supported in *building capacity* to recognize challenges that children may have with participation so that strategies can be introduced in the school right away without the need for formal assessment. An important expectation in this model is that occupational therapists will be a regular presence in schools, available to *collaborate* with teachers, on invitation, right in the classroom. *Coaching* is a specific technique through which the occupational therapist determines what the teacher already knows and builds solutions through collaboratively problem-solving about the reasons for a child’s difficulties, the rationale for trying strategies, modeling the strategies, and supporting their application. Collaborative interactions and observations occur in *context*, wherever the child is experiencing challenges, and strategies are tried out in real time to ensure that they meet the child’s needs (21). These P4C principles result in timely and efficient determination of accommodations and strategies that maximize the participation and inclusion of all children. Families are valued partners who can collaborate with teachers and occupational therapists as needs arise. The family can self-initiate access to the occupational therapist without waiting to be referred, supporting equitable access (22, 23).

As illustrated in Figure 1, the P4C service delivery model uses a tiered approach, in which services are organized in levels or “tiers.” Table 1 describes the tiers. The first tier is foundational and includes *universal* services that are beneficial for all. Services are offered to everyone and help build the capacity of teachers and families to support all children in the school community. Universal services, developed and delivered collaboratively, create a learning environment adaptable and inclusive of children with diverse developmental, communicative, social, and emotional abilities. For children who need additional support, the second level is *targeted*. These services are provided to children who need more support than can be offered through universal services, are usually of short duration, and are often provided in small groups. The third tier is *individualized* and is offered to children who need the most support to participate successfully at school. In contrast with other tiered models, children can receive support simultaneously at all three tiers and, as their needs change, may receive services at any tier depending on need and the classroom environment. Services are provided in the general education setting in partnership and collaboration with teachers. Rather than focusing on “pull out” therapy in which the therapist works with the child in another setting, the therapist works with the child directly in the classroom, on the playground, or in the gymnasium. Unique to P4C, the occupational therapist works closely with teachers across all tiers to problem-solve and jointly identify what services children need and to monitor their responses to the support provided. Because the occupational therapist spends consistent time in the classroom, interacting with children and teachers, children have timely access to services without needing standardized testing, formal identification, or diagnosis.

**Table 1:** Description of Tiers.

Tier	Description of services and supports at each Tier
Tier 3: Individualized services essential for few children	Are intensive and tailored to the individual needs of a child and family Can include direct one-on-one, meditator training, consultation, collaboration, and parent coaching Involve collaboration to support participation and function at home, school and in the community May require referrals to community-based health and social services Include knowledge sharing and capacity building May occur in authentic contexts Tied to curricular goals and use relevant curricular materials.
Tier 2: Targeted services necessary for some children	Are targeted and of greater frequency, intensity, and/or duration; often provided in small groups Involve monitoring response to intervention to determine the need for individual services Include knowledge sharing and capacity building Occur in authentic contexts Tied to curricular goals and use relevant curricular materials.
Tier 1: universal services beneficial for all children	Benefit all children in the classroom, school, and system. Involve promotion of skills foundational to learning, self-care and classroom participation including self-regulation, motor, and productivity skills Involve promotion of positive mental health and physical wellbeing Include knowledge sharing and capacity building Occur in authentic contexts Tied to curricular goals and use relevant curricular materials

This table is adapted from Campbell W., Sahagian Whalen S., Dix L., Pollock N., Jiang A., Kim E., and Missiuna C. (2019). FIRST KIT: resources to support a tiered model of service delivery. Hamilton, ON: CanChild, McMaster University; Available at http://first.machealth.ca/.

## 3. How does Partnering for Change work?

In P4C, the whole school is viewed as the “client.” The occupational therapist’s role involves proactively collaborating with teachers to design physical, social, and learning environments that facilitate the successful participation of all children. Working from a foundation that focuses on relationship-building and mutual sharing of knowledge and expertise, therapists collaborate at Tier 1 with *universal services* to foster inclusion, participation, and skill development in children of all abilities. Specifically, the therapist uses an occupational therapy lens to observe children in classrooms and contexts throughout the school (e.g., cloakroom, hallway, gymnasium, lunchroom, playground), collaborating with the teacher to make changes that will benefit all children. A teacher may also “open the door” to the classroom, inviting the therapist in to address an issue that the educator identifies with some aspect of participation of the whole class.

When implementing universal, class-wide strategies at Tier 1, the occupational therapist and teacher continue to observe and monitor progress. If some children experience challenges following the implementation of universal strategies, they may decide that Tier 2, *targeted services*, are appropriate. This tier involves the occupational therapist collaboratively problem-solving with the teacher, sharing observations, hypothesizing solutions, and potentially trialing new strategies or suggesting ways the teacher might alter activities to match children’s abilities better. The therapist or teacher implements the strategy with smaller numbers of children and monitors their response to intervention over time. If there are children who are still struggling after universal and targeted supports have been provided, then *individualized services* may be necessary. At Tier 3, the therapist collaborates with the teacher to design accommodations and/or modifications to the task or environment for an individual child. This could result in accessing assistive technology, modifying a task to better suit a child’s abilities, or changing the environment to reduce auditory, visual, or social stimulation. When successful strategies are found, they are shared with families to facilitate knowledge transfer to the home environment. The occupational therapist and teacher also consider if and how some of the strategies required for an individual child might be introduced to the entire class to support other children who could benefit.

## 4. When we implemented Partnering for Change, what did we learn?

Our research team has implemented and evaluated Partnering for Change in dozens of schools in Ontario, Canada and collected feedback from teachers, occupational therapists, other health professionals, administrators, and families (18, 19, 22–28). Through over a decade of qualitative and quantitative research, we have learned that when collaboration occurs in the classroom, teacher and occupational therapist capacity is built, children participate more fully, families and administrators are more satisfied, classroom and school environments change, and waitlists for occupational therapy services are eliminated (22, 23, 27). To enable a successful transition to this new way of working, occupational therapists need sufficient time and resources, including training, mentorship, and regular opportunities to share successful approaches and resources with one another (19, 24, 26, 28).

## 5. Discussion

Recently, our team published a realist synthesis of literature that sought to determine when, why, for whom and under what circumstances tiered models of rehabilitation services, such as P4C, are successful in educational settings (29). Following analysis of 52 peer-reviewed articles from occupational therapy, speech-language pathology and physiotherapy, several features were identified within the broader context of school-based rehabilitation services that facilitated successful outcomes of tiered approaches, including: (1) the belief that children with disabilities can and should learn in inclusive environments; and (2) the need for universally designed curricula that promote access and participation for all children.

Additionally, this synthesis of the literature identified three processes that rehabilitation professionals needed to focus on when delivering tiered services: fostering *collaborative* relationships, *building capacity* for all, and providing authentic services *in context*. While articles discussing P4C were represented among the reviewed literature, these articles were a subset of a larger pool of international evidence. Thus, it is validating to have learned that these processes had already been named as principles of P4C, providing further evidence in support of the model. Even more exciting is that new research is emerging to demonstrate that when in-service occupational therapists and educators engage in joint professional development about collaboration, their self-perceived knowledge and skills are enhanced and behaviors indicative of richer interprofessional collaboration are observed (30). Thus, future research can explore not only what principles are central to P4C, but also how to ensure they are actualized in practice.

With respect to broader adoption of P4C, Meuser and colleagues (21) studied the P4C model in four Dutch and two Swedish elementary schools and determined that the model facilitated collaboration and enhanced children’s inclusion and participation (21). This finding supports prior studies of P4C as well as the realist synthesis (29). Further, we are constructing a detailed explanation of how, when, why, and for whom P4C “works” so that we can enable others to adopt and adapt P4C to their unique circumstances in ways that promote success and positive outcomes for all (29).

Increased adoption of tiered models, such as P4C, in school-based services has been shown to facilitate increased children’s participation and inclusion. In turn, children’s increased engagement in school has been shown to increase academic success and social engagement for children with disabilities (4, 31) and their peers (7). We have a responsibility to continue the movement toward tiered school-based services to support every child’s achievement, inclusion, and sense of belonging at school.

Inclusive education is not just an aspiration. It is a global imperative. In our experience, nearly all teachers, when given the opportunity, will choose to invite occupational therapists into their classrooms, reflecting their openness to this new role. Collaborations between occupational therapists and teachers in the general education classroom provide equitable and earlier access to supports for all children, including for children who have disabilities as well as for children whose circumstances place them at-risk. By collaborating with teachers, maintaining a consistent presence in the school, and serving the whole school community, occupational therapists can adopt this practice to support inclusive education and foster children’s successful participation at school.

## Data availability statement

The original contributions presented in the study are included in the article, further inquiries can be directed to the corresponding author.

## Author contributions

WC: Conceptualization, Data curation, Formal analysis, Funding acquisition, Investigation, Methodology, Project administration, Supervision, Validation, Visualization, Writing – original draft. CM: Conceptualization, Data curation, Formal analysis, Funding acquisition, Investigation, Methodology, Project administration, Supervision, Validation, Visualization, Writing – original draft. LD: Conceptualization, Data curation, Formal analysis, Investigation, Project administration, Visualization, Writing – original draft. SW: Conceptualization, Data curation, Formal analysis, Methodology, Visualization, Writing – original draft.
